# First-Line Immune Checkpoint Inhibitors in Older Patients with Advanced Urothelial Carcinoma: A Systematic Review and Meta-Analysis

**DOI:** 10.3390/cancers18182924

**Published:** 2026-09-09

**Authors:** Dalila Incognito, Andrea D’Arienzo, Sergio Facchini, Chiara Barraco, Antonio Picone, Antonella Nicastro, Giuliana Ciappina, Giordana Di Mauro, Antonio Ieni, Massimiliano Berretta, Gaetano Facchini

**Affiliations:** 1 Medical Oncology Unit, Department of Human Pathology “G. Barresi”, School of Specialization in Medical Oncology, University of Messina, 98122 Messina, Italy; incognitodalila@gmail.com (D.I.); giordana.di.mauro@hotmail.it (G.D.M.); 2 Translational Molecular Medicine and Surgery, Department of Clinical and Experimental Medicine, University of Messina, 98122 Messina, Italy; 3 Oncology Unit, “Santa Maria Delle Grazie” Hospital, 80078 Pozzuoli, Italy; andrea.darienzo@aslnapoli2nord.it (A.D.); chiara.barraco@aslnapoli2nord.it (C.B.); antonella.nicastro@aslnapoli2nord.it (A.N.); 4 Experimental Clinical Abdominal Oncology Unit, Istituto Nazionale Tumori-IRCCS-Fondazione G. Pascale, 80131 Naples, Italy; sergio.facchini@istitutotumori.na.it; 5 Department of Clinical and Experimental Medicine, University of Messina, 98122 Messina, Italy; antonio.picone@polime.it (A.P.); mberretta@unime.it (M.B.); 6 Section of Experimental Medicine, Department of Medical Sciences, University of Ferrara, 44121 Ferrara, Italy; giuliana.ciappina@unife.it; 7 Applied Biology and Experimental Medicine, Department of Chemical, Biological, Pharmaceutical and Environmental Sciences, University of Messina, 98166 Messina, Italy; 8 Department of Human Pathology of Adult and Childhood “Gaetano Barresi”, University of Messina, 98121 Messina, Italy; antonio.ieni@unime.it; 9 Division of Medical Oncology, “G. Martino” University Hospital, University of Messina, 98124 Messina, Italy

**Keywords:** immunotherapy, metastatic urothelial cancer, older patients

## Abstract

Urothelial carcinoma predominantly affects older patients, who often exhibit frailty, comorbidities, renal impairment, and reduced physiological reserve. We evaluated overall survival (OS) and progression-free survival (PFS) with first-line immune-based strategies in patients aged ≥65 years with advanced or metastatic urothelial carcinoma. The aim was to characterize treatment effects within this age-defined subgroup, not to determine whether age modifies treatment efficacy compared with younger patients. **Methods**: We conducted a systematic review and meta-analysis of phase 3 randomized trials published from January 2015 to May 2026 reporting age-specific survival outcomes. Combination regimens were compared with platinum-based chemotherapy using random-effects models. **Results**: Combination strategies improved OS versus chemotherapy (HR 0.79, 95% CI 0.64–0.96), although heterogeneity was substantial (I^2^ = 69.7%) and the prediction interval crossed the null (0.48–1.27). Class-specific HRs were 0.56 (95% CI 0.26–1.20) for ADC plus ICI, 0.85 (0.77–0.95) for ICI plus chemotherapy, and 0.91 (0.34–2.42) for dual-checkpoint inhibition. PFS favoured experimental treatment but remained imprecise (HR 0.54, 95% CI 0.27–1.08; I^2^ = 89.5%). Excluding ADC plus ICI trials attenuated the OS effect (HR 0.88, 95% CI 0.80–0.96), indicating that the pooled estimate was influenced by these trials. ICI monotherapy did not improve OS. **Conclusions**: First-line immune-based combinations improved survival in selected patients aged ≥65 years, but benefit varied across treatment classes and should not be considered uniform across regimens. These findings do not establish whether age modifies treatment efficacy. Age-specific safety data were insufficient to define the benefit–risk profile of individual strategies.

## Simple Summary

This study summarizes age-specific efficacy data from randomized trials of first-line immune-based treatments in patients aged ≥65 years with advanced urothelial carcinoma. Combination regimens improved overall survival compared with platinum-based chemotherapy, but the size of the benefit differed across treatment strategies and should not be considered uniform across regimens. Progression-free survival generally favoured experimental treatments, although estimates were affected by substantial heterogeneity. Immune checkpoint inhibitor monotherapy did not improve overall survival in the available ≥65-year subgroups. These results describe treatment efficacy in older patients but do not show whether age modifies treatment benefit compared with younger patients. Age-specific safety and treatment-discontinuation data were reported inconsistently, so the overall benefit–risk profile of individual strategies could not be assessed. Treatment choice in older patients should therefore consider the specific regimen, comorbidities, functional status, and expected toxicity.

## 1. Introduction

Bladder cancer (BC) is a common malignancy worldwide [1,2]. Population ageing is expected to increase BC incidence, making the management of older patients increasingly relevant [3,4]. BC is predominantly a disease of advanced age, with a mean age at diagnosis of approximately 73 years and nearly 80% of cases occurring in individuals older than 65 years [5]. Moreover, increasing age at diagnosis has consistently been associated with significantly worse overall survival and BC-specific survival, even in the era of more effective systemic therapies [1,6]. For patients with advanced urothelial carcinoma (UC), platinum-based chemotherapy (CT) has represented the cornerstone of first-line treatment for several decades, with cisplatin-based combinations remaining the preferred option for eligible patients [7,8]. However, the clinical management of older individuals is frequently complicated by frailty, impaired renal function, comorbidities, and reduced physiological reserve, all of which substantially limit eligibility for cisplatin-based regimens [9]. Older patients are often under-represented in randomized clinical trials and may receive less intensive treatments than younger individuals, despite the potential benefit of standard therapeutic approaches [4]. The high mutational burden and intrinsic immunogenicity of UC have provided a strong biological rationale for the development of immune-based therapeutic strategies [10,11]. Ageing is accompanied by immunosenescence, a complex process characterized by impaired immune surveillance, reduced cytotoxic activity, and chronic low-grade inflammation, all of which may influence cancer progression and treatment outcomes; T-cell dysfunction and the establishment of an immunosuppressive microenvironment can modify antitumor immune responses [12,13]. In recent years, the introduction of ICI targeting the PD-1/PD-L1 axis has profoundly changed the therapeutic landscape of advanced UC, demonstrating durable clinical responses and establishing immunotherapy as a key component of treatment algorithms [14]. Furthermore, available evidence suggests that immunotherapy may provide comparable efficacy and tolerability across different age groups, supporting its use even in older populations who are frequently unsuitable for platinum-based CT [4].

Despite these advances, the magnitude of benefit derived from first-line and maintenance immune-based strategies in older patients remains incompletely defined. Older adults are often under-represented or clinically selected in pivotal trials, and age-specific outcomes are frequently reported only in exploratory subgroup analyses [4,14]. Therefore, a comprehensive synthesis of the available evidence is needed to characterize treatment effects in older trial participants. The aim of the present systematic review and meta-analysis was to estimate the efficacy of first-line immune-based strategies in older patients with advanced or metastatic urothelial carcinoma, using the age categories reported in the included trials. Maintenance studies were summarized descriptively. The analysis was designed to evaluate treatment effects within older age-defined subgroups, rather than to determine whether chronological age modifies treatment efficacy. Assessment of treatment-effect modification by age would require direct comparison of treatment effects across age groups and formal treatment-by-age interaction testing.

## 2. Materials and Methods

### 2.1. Protocol Registration and Endpoints

This systematic review and meta-analysis was conducted according to the Preferred Reporting Items for Systematic Reviews and Meta-Analyses (PRISMA) 2020 guidelines [15]. The protocol was registered in PROSPERO with the number CRD420261430472 (version 2.0); protocol deviations and checklist are provided in the Supplementary Sections S1 and S3).

The primary objective was to evaluate the efficacy of first-line immune-based combination strategies in older patients with advanced or metastatic UC. Overall survival (OS) was the primary endpoint, and progression-free survival (PFS) was the secondary endpoint. The analysis was designed to estimate treatment effects within the older age-defined subgroup reported in the included trials, rather than to assess treatment-by-age interaction or formally compare efficacy between older and younger patients.

### 2.2. Eligibility Criteria

We searched for phase 3 randomized controlled trials evaluating first-line immunotherapy, either alone or in combination, in advanced or metastatic UC compared with platinum-based chemotherapy. Trials were eligible if they reported hazard ratios (HRs) and 95% confidence intervals (CIs) for OS and/or PFS in an older age-defined subgroup. Because there is no universally accepted chronological threshold defining an older patient, and pivotal trials most commonly report age-specific outcomes using a 65-year cutoff [16], patients aged ≥65 years were considered the primary older subgroup for this meta-analysis. This threshold was adopted as an operational definition based on available trial reporting, rather than as a clinical or geriatric definition of elderly or frail patients. For terminological consistency, the term “older patients” refers to patients aged ≥65 years throughout the manuscript, unless a different age threshold is explicitly specified. Additional age strata, when available, are reported using the thresholds provided in the original trials, such as 65–74 years or ≥75 years. This age-based definition should not be interpreted as a surrogate for frailty, comorbidity burden, renal function, performance status, physiological reserve, or very old age. Eligibility criteria were based on the PICO (Population, Intervention, Comparison, Outcome) framework (see Supplementary Sections S1 and S2). The exclusion criteria were as follows: phase I or single-arm phase II trials; reviews, meta-analysis or case reports; non-randomized, retrospective, real-world or later-line trials; lacking of age-specific efficacy outcomes for patients aged ≥65 years or studies reporting safety or quality of life as a primary outcome; studies evaluating locoregional therapies (i.e., radiotherapy or surgery) or treatment other than immunotherapy; studies evaluating non-UC or non-metastatic UC, in vitro or animal trials.

### 2.3. Search Strategy and Study Selection

The following string was used to query the PubMed/MEDLINE, Cochrane Central Register of Controlled Trials and Embase databases from Jan 2015 to May 2026: (“metastatic” OR “advanced” OR “unresectable”) AND “urothelial” AND (“untreated” OR “first line” OR “maintenance”). A manual search of abstracts from major international oncology congresses, including the American Society of Clinical Oncology (ASCO) and the European Society for Medical Oncology (ESMO), was also performed. Two independent reviewers (D.I. and A.D.) screened the retrieved records and applied the pre-specified inclusion and exclusion criteria using Rayyan software (Rayyan Systems Inc., Cambridge, MA, USA; https://www.rayyan.ai/) [17]. Any controversies were resolved by consultation with supervisors (M.B. and G.F.). The references reported in each selected study were further manually inspected in order to retrieve potential undetected manuscripts.

### 2.4. Data Extraction and Risk of Bias

These data were extracted by two independent reviewers (D.I. and A.D.) and collected in an electronic spreadsheet: study name and year, experimental arm, control arm, median follow-up, number of patients enrolled, number and proportion of patients aged ≥65 years, median age, ECOG performance status, cisplatin eligibility, sites of metastasis, median follow-up, median OS and PFS for overall and ≥65-year populations, HR for OS and PFS in overall and ≥65-year populations, and lower and upper 95% CI for HRs.

Two independent reviewers (D.I. and A.D.) assessed the risk of bias of the age-specific outcome results from the included randomized controlled trials using the Cochrane Risk of Bias 2 (RoB 2) tool. Disagreements were resolved through discussion and, when necessary, consultation with two senior reviewers (M.B. and G.F.). The certainty of the evidence for each outcome was assessed using the Grading of Recommendations Assessment, Development and Evaluation (GRADE) approach and rated as high, moderate, low, or very low [18].

### 2.5. Statistical Data Analysis

The HRs and 95% CIs for OS and PFS were used as measures of effect. For trials reporting outcomes in fractionated age brackets (e.g., 65–74 years and ≥75 years) without providing a single overall estimate for patients aged ≥65 years, a trial-level pooled HR for the broader ≥65 years population was pre-calculated through the inverse-variance weighting method on the log-transformed HRs. These derived estimates were considered approximations of the treatment effect in the overall ≥65-year population and were also explored in sensitivity analyses excluding reconstructed subgroup estimates. To evaluate the pooled efficacy of first-line treatments, a standard pairwise meta-analysis was conducted using a random-effects model. The restricted maximum likelihood (REML) estimator was utilized to calculate the between-study variance (τ^2^); 95% CIs for the pooled effect estimates were computed using the Hartung–Knapp (HK) method. For pooled first-line analysis, 95% prediction intervals were also generated. Statistical heterogeneity was evaluated using the Cochran’s Q test and the I^2^ statistic, applying a significance threshold of *p* < 0.10. I^2^ values were interpreted as low (0–25%), moderate (25–50%), and high (50–100%). To assess the summary effect of distinct therapeutic strategies in the first-line setting, experimental combination regimens were pooled against standard chemotherapy and also stratified by pharmacological class. Handling multi-arm trials that included both ICI monotherapy and combination regimens required pre-specified analytical decisions. To avoid unit-of-analysis errors and double-counting of participants in the shared CT control arm, the primary pairwise meta-analysis was restricted to combination versus standard CT. For treatment strategies where formal quantitative synthesis was methodologically precluded due to violations of inter-trial independence, efficacy outcomes were summarized descriptively.

Potential effect modifiers, including ECOG performance status, visceral metastases, and prior platinum-based CT, were compared across trials. Since baseline clinical characteristics specific to older patients were unavailable, demographic and clinical data from the overall intention-to-treat populations were used as a representative estimate.

All statistical analyses were performed using RStudio (version 2026.07.1), primarily utilizing the meta package. The results were considered statistically significant when the corresponding 95% CIs excluded the null value or when the two-sided *p* value was <0.05.

### 2.6. Sensitivity Analyses

To test the robustness of our findings and explore potential sources of methodological or clinical heterogeneity, a comprehensive series of sensitivity analyses was pre-planned. Specifically, the following analyses were performed: (1) leave-one-out sensitivity analysis to identify potential outliers and determine if the summary effect was driven by any single trial; (2) subgroup analysis for trial design (multi-arm vs. two arms); (3) comparing outcomes between the subgroups of ≥65, 65–74 and ≥75 years old.

## 3. Results

### 3.1. Study Selection

**Figure 1:**
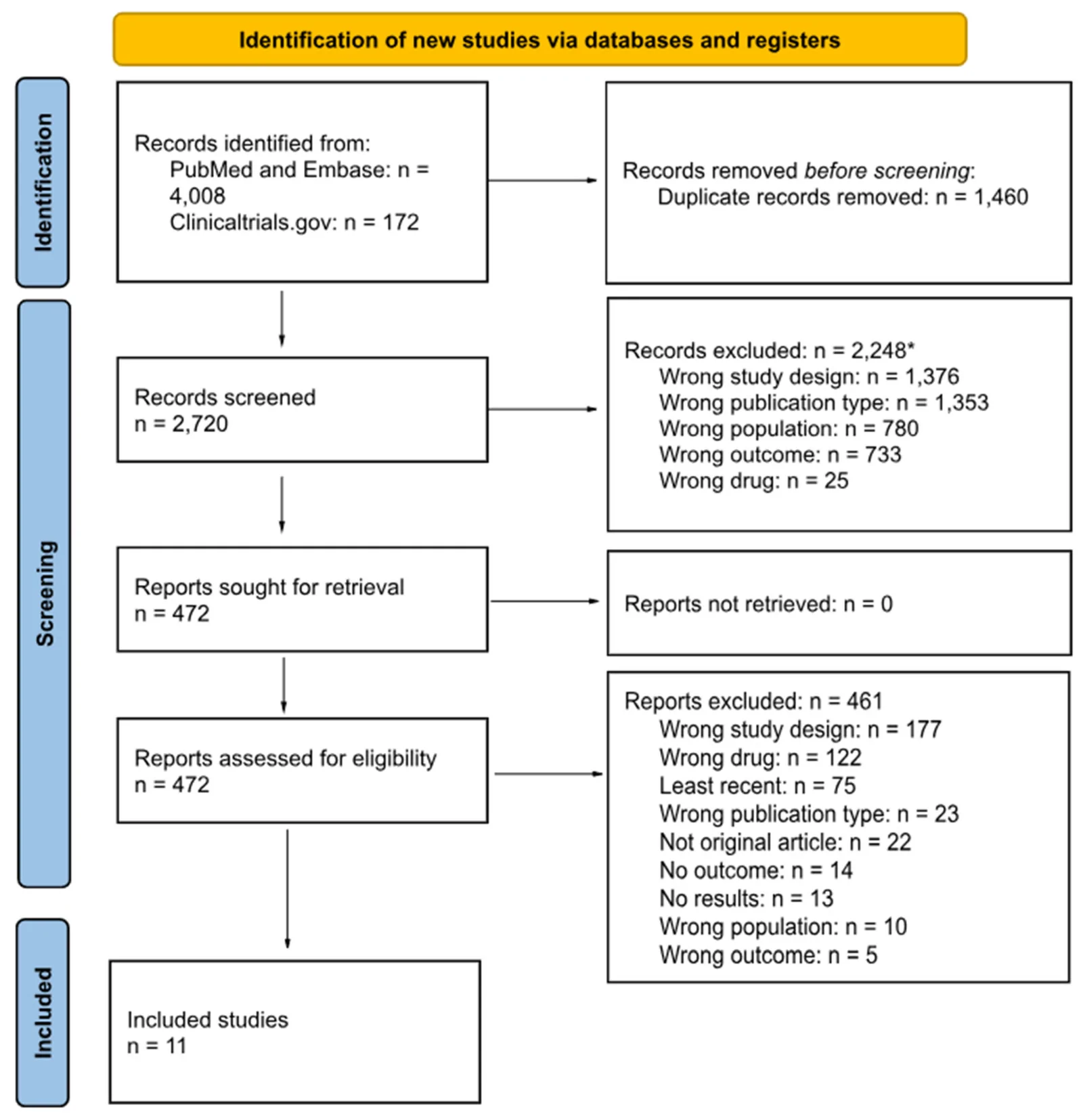
PRISMA flow diagram of study selection. The database and registry searches identified 4180 records, of which 1460 duplicates were removed. After screening 2720 records, 472 reports underwent full-text assessment. Of these, 461 were excluded for the reasons shown, resulting in 11 reports corresponding to eight unique studies included in the systematic review. * More than one exclusion reason could be assigned to the same record during screening.

A total of 4180 records were identified. After removing duplicates and applying the inclusion and exclusion criteria, 472 reports underwent full-text assessment. A total of eleven reports were selected for data extraction, corresponding to eight unique trials: six first-line trials and two maintenance trials. The most common exclusion reasons during full-text analysis were as follows: wrong study design (37.5%), wrong drug (26%) and less mature report of an already included trial (i.e., least recent; 16%). Notably, during full-text analysis, the BAYOU trial [19] was excluded because its control regimen did not meet the pre-specified comparator criterion for the first-line analysis (Figure 1, PRISMA diagram).

The two maintenance treatment-identified trials were JAVELIN Bladder 100 [20] and JAVELIN Bladder Medley [21]. Comparison was not performed because the efficacy analysis of JAVELIN Bladder Medley incorporated propensity score-weighted external data from JAVELIN Bladder 100 to extend the avelumab control arm, precluding the independence of effect estimates. Therefore, maintenance treatment results are presented exclusively as a narrative synthesis.

### 3.2. Study and Patient Characteristics

Six randomized trials evaluating first-line immune-based treatment strategies were included, of which four multi-arm (Checkmate 901 arm A [22] and C [23], KEYNOTE-361 arm A and B [24], DANUBE arm A and B [25], IMvigor130 arm A and C [26]) and two two-arm randomized trials (EV-302 [27] and RC48-C016 [28]). Overall, ten experimental treatment arms were identified: two ADC + ICI combinations (EV-302 [27] and RC48-C016 [28]), three ICI + CT regimens (KEYNOTE-361 arm A [24], CheckMate 901 arm C [23] and IMvigor130 arm A [26]), three ICI monotherapy arms (KEYNOTE-361 arm B [24], DANUBE arm B [25] and IMvigor130 arm C [26]), and two dual-ICI combinations (CheckMate 901 arm A [22], DANUBE arm A [25]). Monotherapy arms from KEYNOTE-361 [24] and IMvigor130 [26] were excluded to avoid the double-counting of shared control arm, while the two CheckMate 901 comparisons arms [22,23] were retained because they had separate control cohorts. The median follow-up of first-line included trials was 31.7 months (interquartile range 23,65–37,40). All six studies reported OS outcome for patients aged ≥65 years. Age-specific HRs for ICI monotherapy were available for only two trials (KEYNOTE-361 [24] and IMvigor130 [26] evaluating pembrolizumab and atezolizumab, respectively) and were summarized descriptively; the durvalumab monotherapy arm of DANUBE [25] did not report an age-specific HR and was not included in the efficacy synthesis. A PFS age-specific HR was reported in four trials (CheckMate 901 arm C [23], EV-302 [27], IMvigor130 arm A [26] and RC48-C016 [28]). Finally, seven combination vs. CT comparisons contributed to the primary pairwise meta-analysis: enfortumab vedotin + pembrolizumab, disitamab vedotin + toripalimab, pembrolizumab + CT, nivolumab + CT, atezolizumab + CT, ipilimumab + nivolumab and durvalumab + tremelimumab. Control regimens consisted of platinum-based CT, although the CT backbone and eligibility for cisplatin varied across trials (Table 1 and Table 2, studies and patients’ characteristics).

Across randomized participants, 3369 patients were allocated to experimental and 2309 to control regimens; 1576 (46.7%) and 1102 (47.7%) patients, respectively, were aged ≥65 years. All trials had a median age of ≥65 years in overall enrolled populations; only CheckMate 901 arm A [22] had a median age of ≥70 years. Although the EV-302 [27] publication reported baseline age categories using a ≥75-year threshold, HRs for both OS and PFS were reported for patients aged ≥65 years and were used for the present analyses. Detailed baseline clinical characteristics specific to the ≥65-year subgroup were largely unavailable across the included trials. Therefore, the demographic and clinical characteristics reported in Table 1 and Table 2 predominantly refer to the overall intention-to-treat populations and were used only to contextualize the populations enrolled in each trial. Baseline characteristics were generally balanced within trials. However, clinically relevant differences were observed across studies. RC48-C016 [28] was the only trial restricted to a HER2-positive population and included a high proportion of patients with ECOG performance status 1 (about 73%). In CheckMate 901 [22,23], the dual-ICI and ICI + CT comparisons enrolled populations with distinct cisplatin-eligibility profiles, with arm A restricted to cisplatin-ineligible patients and arm C enrolling cisplatin-eligible patients.

Two maintenance trials were identified. JAVELIN Bladder 100 [20] evaluates, in patients non-progressing from platinum-based first-line CT, avelumab maintenance versus best supportive care (BSC); this trial reported the proportion of patients aged ≥65 years in both treatment groups. The JAVELIN Bladder Medley [21] evaluates different avelumab-based combinations vs. avelumab + BSC; this trial did not report detailed baseline characteristics for the older subgroup, although an age-stratified PFS HR was available.

### 3.3. RoB

Risk of bias was assessed using the Cochrane RoB 2 tool [18] and summarized in Supplementary Figure S1. EV-302 [27], CheckMate 901 arm C [23], KEYNOTE-361 [24], IMvigor130 [26], and DANUBE [25] were judged to be at low overall risk of bias. Some concerns were identified for RC48-C016 [28] because allocation concealment could not be fully verified, and for CheckMate 901 arm A [22] and JAVELIN Bladder 100 [20] because of limited information on the pre-specification of the age-specific analyses. JAVELIN Bladder Medley [21] was considered at high overall risk of bias because the efficacy estimate incorporated a propensity score-weighted external control, with additional concerns related to missing outcome data, investigator-assessed outcome measurement, and selection of the reported result. Publication bias was not formally tested due to the total number of studies enrolled (*n* = 7).

### 3.4. Efficacy Analysis

#### 3.4.1. First-Line Treatment and OS

**Figure 2:**
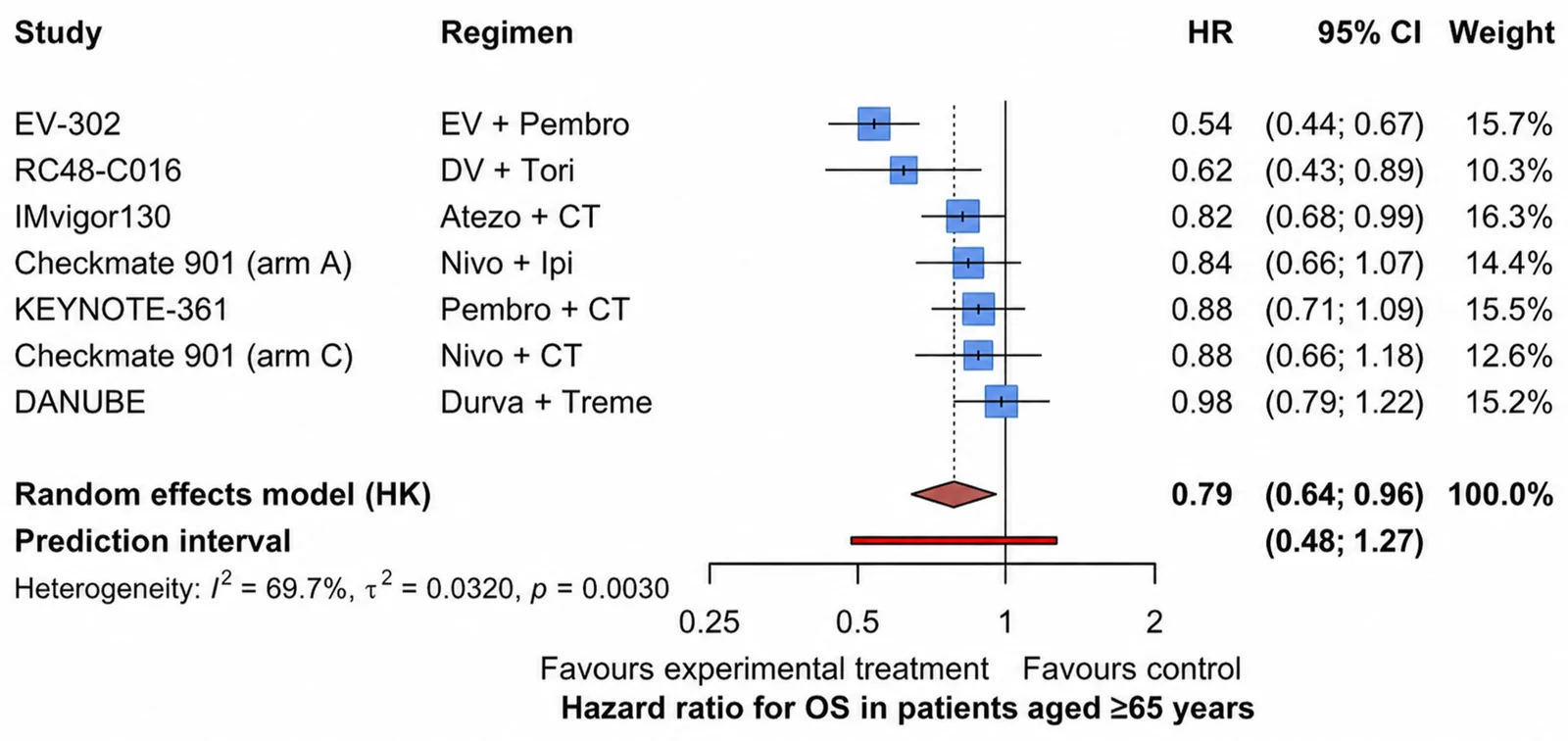
Overall survival with first-line immune-based combinations in patients aged ≥65 years with advanced urothelial carcinoma. Forest plot of hazard ratios for overall survival among patients aged ≥65 years treated with first-line immune-based combination regimens versus platinum-based chemotherapy. Study-specific estimates were pooled using a random-effects model with restricted maximum likelihood estimation and Hartung–Knapp adjustment. Squares represent study-specific hazard ratios, with their size proportional to study weight; horizontal lines indicate 95% confidence intervals, and the diamond represents the pooled estimate.

**Figure 3:**
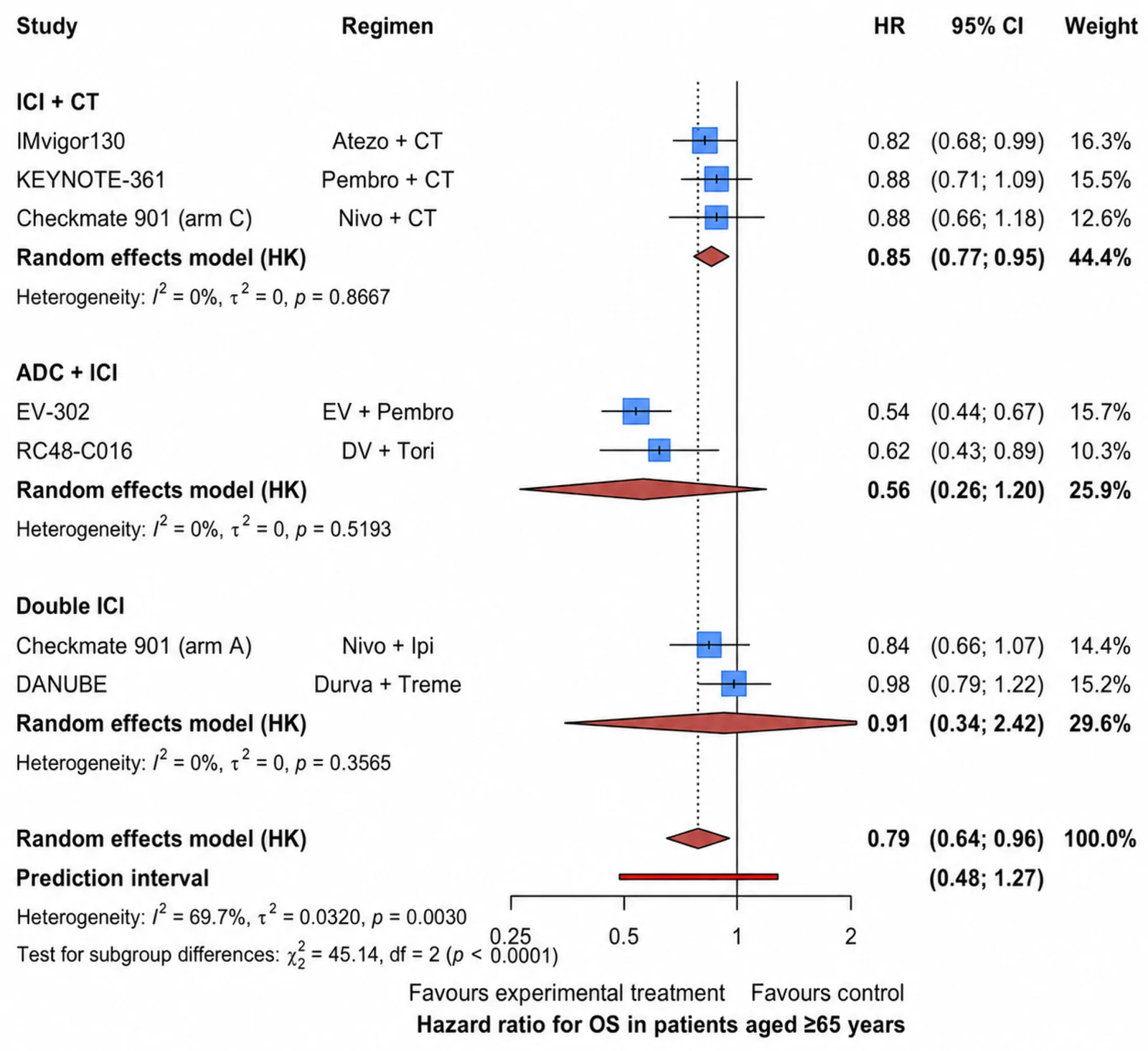
Overall survival according to the class of first-line immune-based combination in patients aged ≥65 years with advanced urothelial carcinoma. Forest plot of hazard ratios for overall survival among patients aged ≥65 years, stratified by treatment class. Study-specific estimates were pooled using random-effects models with restricted maximum likelihood estimation and Hartung–Knapp adjustment. Squares represent study-specific estimates, with their size proportional to study weight; horizontal lines indicate 95% confidence intervals, and diamonds represent pooled estimates.

The primary meta-analysis included seven first-line combination-versus-platinum-based CT comparisons encompassing clinically distinct therapeutic strategies. In the overall random-effects model, combination regimens were associated with improved OS in patients aged ≥65 years compared with chemotherapy (HR 0.79, 95% CI 0.64–0.96) with high between-study heterogeneity (I^2^ = 69.7%; τ^2^ = 0.0320; *p* = 0.0030). The 95% prediction interval ranged from 0.48 to 1.27 (Figure 2). Given the expected heterogeneity across treatment strategies, a pre-specified stratified analysis was performed (Figure 3). Treatment regimens were grouped as DUAL ICI, ADC plus ICI, and ICI plus CT. Pooled HRs were 0.91 (95% CI 0.34–2.42) for DUAL ICI combinations, 0.56 (95% CI 0.26–1.20) for ADC + ICI, and 0.85 (95% CI 0.77–0.95) for ICI + CT. The estimated heterogeneity within each treatment category was I^2^ = 0%; however, the small number of comparisons contributing to each subgroup limits the reliability of these estimates and should not be interpreted as evidence of absence of underlying heterogeneity. The test for subgroup differences was statistically significant (χ^2^ = 45.14; *p* < 0.0001). However, given the limited number of comparisons and the substantial clinical and methodological differences between the included regimens and trial populations, this finding should be considered exploratory and should not be interpreted as definitive evidence of differential efficacy between treatment classes.

Age-specific hazard ratios for ICI monotherapy versus CT were available for only two trials. In KEYNOTE-361 [24], pembrolizumab monotherapy did not improve OS compared with CT in patients aged ≥65 years (HR 1.15, 95% CI 0.84–1.59). In IMvigor130 [26], inverse-variance pooling of the 65–79 and ≥80 years subgroups yielded an estimated HR of 0.98 (95% CI 0.79–1.21) for atezolizumab versus CT in patients aged ≥65 years. Neither study showed a statistically significant OS benefit with ICI monotherapy in the available ≥65-year subgroups. Also, an exploratory analysis according to the study design is provided in Supplementary Figure S2.

#### 3.4.2. First-Line Treatment and PFS

**Figure 4:**
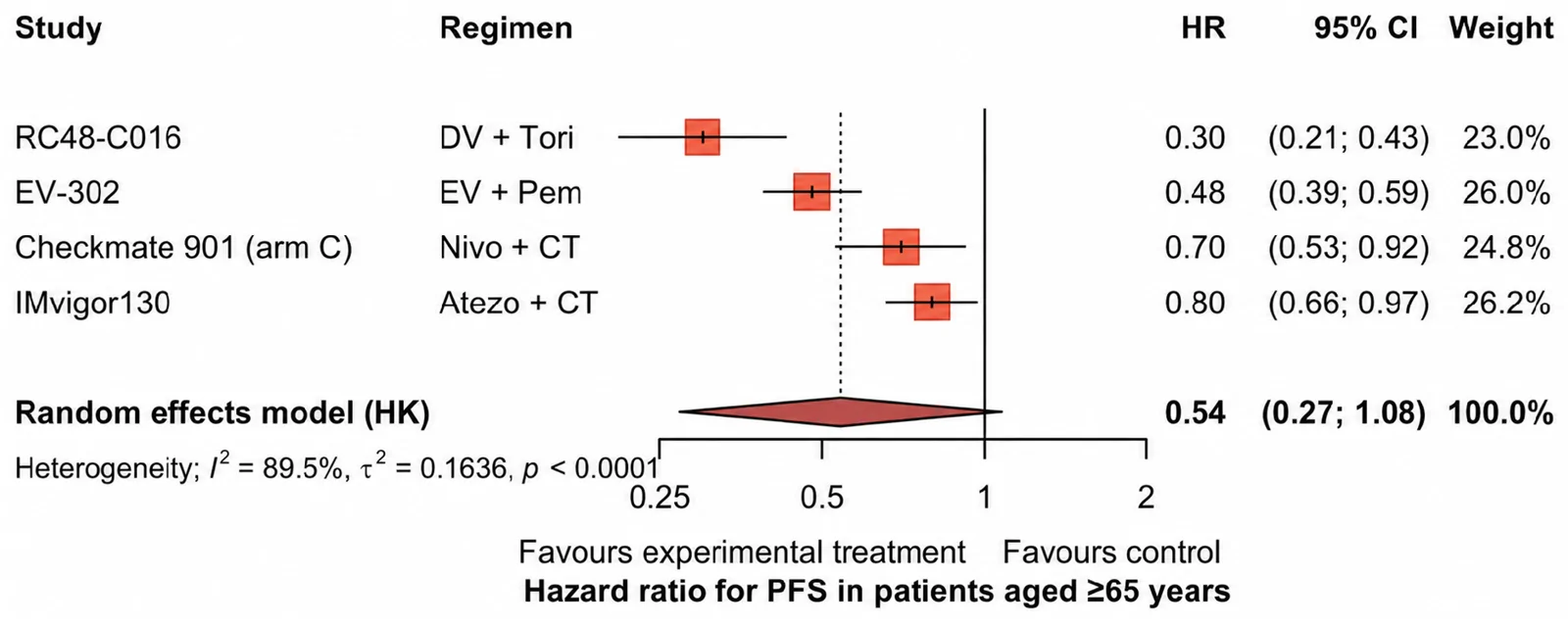
Progression-free survival with first-line immune-based combinations in patients aged ≥65 years with advanced urothelial carcinoma. Forest plot of hazard ratios for progression-free survival among patients aged ≥65 years treated with first-line immune-based combination regimens versus platinum-based chemotherapy. Squares represent study-specific estimates, with their size proportional to study weight; horizontal lines indicate 95% confidence intervals, and the diamond represents the pooled estimate.

The PFS outcomes for the ≥65-year subgroup were available for four comparisons (RC48-C016 [28], EV-302 [27], CheckMate 901 arm C [23], and IMvigor130 [26]), comprising 1440 patients in the experimental arms and 1389 in the control arms (48% and 47.3% of patients aged ≥65 years, respectively). All individual comparisons favoured the experimental regimen and demonstrated a statistically significant PFS benefit, with HRs ranging from 0.30 to 0.80. However, the pooled random-effects estimate did not reach formal statistical significance (HR 0.54, 95% CI 0.27–1.08), with high between-study heterogeneity (I^2^ = 89.5%; τ^2^ = 0.1636; *p* < 0.0001) (Figure 4). Although the direction of benefit was consistent across studies, its magnitude varied considerably, likely reflecting the clinical and therapeutic heterogeneity of the included regimens, particularly the inclusion of both ADC-based combinations and ICI–chemotherapy strategies. Overall, these findings suggest a potential PFS benefit in patients aged ≥65 years, but the pooled estimate remains imprecise and should be interpreted cautiously.

#### 3.4.3. Maintenance Treatment: Descriptive Synthesis

The OS data for the ≥65-year subgroup were not available for the avelumab plus sacituzumab govitecan combination. In JAVELIN Bladder 100, avelumab maintenance plus BSC improved PFS versus best supportive care in patients aged ≥65 years (HR 0.46, 95% CI 0.37–0.57). Conversely, the addition of sacituzumab govitecan to the avelumab in the JAVELIN Bladder Medley trial showed a numerically lower risk of progression or death than avelumab alone although not reaching formal statistical significance (HR 0.68, 95% CI 0.41–1.14).

#### 3.4.4. Exploratory and Sensitivity Analyses

**Figure 5:**
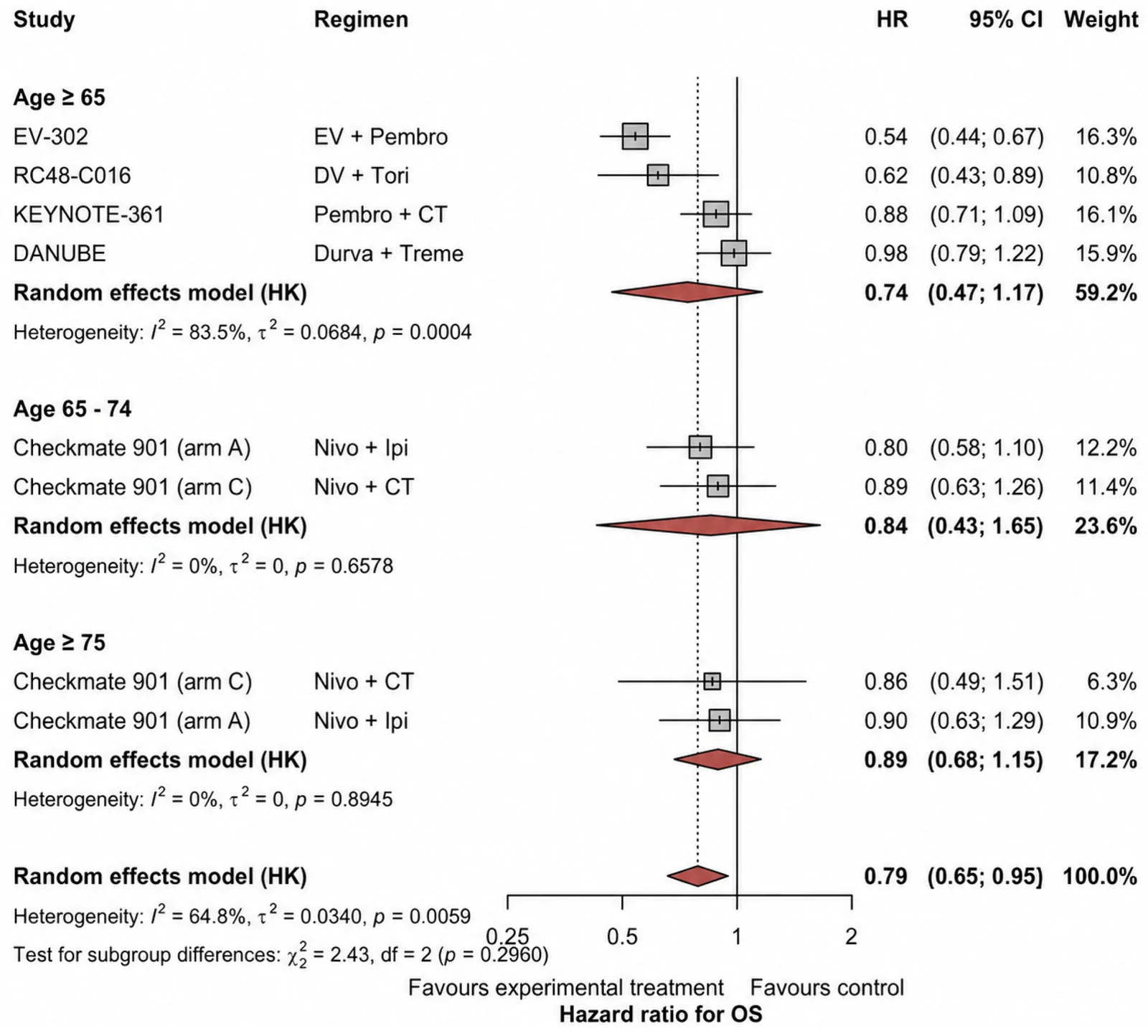
Overall survival with first-line immune-based combinations according to age subgroup. Forest plot of hazard ratios for overall survival stratified according to the age categories reported in the individual trials. These exploratory findings should be interpreted cautiously because age categories and contributing trials differed across subgroups. Squares represent study-specific estimates, with their size proportional to study weight; horizontal lines indicate 95% confidence intervals, and diamonds represent pooled estimates.

OS across older age strata was evaluated as pre-specified sensitivity analysis. This analysis was intended to describe treatment-effect estimates within the reported age strata and was not designed to assess treatment-by-age interaction. The pembrolizumab monotherapy arm of KEYNOTE-361 [24] and the IMvigor130 [26] trial were excluded to avoid the unit-of-analysis errors arising from the double-counting of the shared chemotherapy control group. In the ≥65 years old population, the pooled HR for the included combination regimens was 0.74 (95% CI, 0.47–1.17), with high heterogeneity (I^2^ = 83.5%, τ^2^ = 0.0684, *p* = 0.0004) consistent with the variable magnitude of effect observed across ADC-based and other immune-based regimen (Figure 5). In the 65–74-year subgroup, the random-effect estimate did not demonstrate a statistically significant OS advantage for the experimental combinations over standard CT (HR 0.84; 95% CI, 0.43–1.65). Similarly, the ≥75 years subgroup yielded a pooled HR of 0.89 (95% CI, 0.68–1.15). These age-stratified estimates should be interpreted cautiously because the contributing trials and treatment strategies differed across age categories.

Pre-specified leave-one-out analyses were performed and are reported in Supplementary Table S1. A separate sensitivity analysis excluding ADC plus ICI combinations was also conducted and is reported in Supplementary Figure S3. After exclusion of ADC plus ICI combinations, the direction of effect remained favourable, although the pooled OS estimate was attenuated to HR 0.88 (95% CI, 0.80–0.96). The test for subgroup differences between dual ICI and ICI plus CT combinations was not statistically significant (χ^2^ = 0.73; *p* = 0.3944), providing no evidence that the average treatment effects differed between these two treatment-strategy categories at the trial level.

### 3.5. Certainty of Evidence

The GRADE assessment is reported in Supplementary Table S2. Overall, the certainty of evidence was rated as low for OS with first-line combination strategies versus platinum-based chemotherapy, mainly because of exploratory age-specific subgroup reporting and substantial inconsistency across clinically distinct treatment strategies. Certainty was rated as very low for PFS because of substantial heterogeneity and imprecision. Evidence for ICI monotherapy and maintenance strategies was summarized descriptively and was considered very low because of the limited number of available age-specific estimates, incomplete reporting of subgroup-specific safety data, and methodological limitations precluding quantitative indirect comparison.

## 4. Discussion

This systematic review and meta-analysis examined age-specific efficacy outcomes with first-line immune-based strategies in patients aged ≥65 years with advanced urothelial carcinoma. First-line combination regimens were associated with improved OS compared with platinum-based chemotherapy (HR 0.79, 95% CI 0.64–0.96). However, between-study heterogeneity was high and the prediction interval crossed the null value. Therefore, the overall pooled estimate should be interpreted as an aggregate summary across heterogeneous interventions, rather than as evidence of a uniform treatment effect across all immune-based combinations. Treatment-class-specific analyses were more informative for clinical interpretation. The most favourable numerical estimate was observed for ADC plus ICI combinations (HR 0.56, 95% CI 0.26–1.20), followed by ICI plus chemotherapy (HR 0.85, 95% CI 0.77–0.95) and dual ICI combinations (HR 0.91, 95% CI 0.34–2.42). However, these estimates were based on a limited number of comparisons, had wide confidence intervals, and were derived from trials differing in design, eligibility criteria, biomarker selection, cisplatin eligibility, and treatment composition. Accordingly, class-specific analyses should be considered exploratory and cannot establish the superiority of one therapeutic strategy over another. The attenuation of the pooled OS estimate after exclusion of ADC plus ICI combinations (HR 0.88, 95% CI 0.80–0.96) further indicates that the magnitude of the overall effect was mostly influenced by the larger treatment effects observed in the ADC-based trials. Evidence for ICI monotherapy was limited. In KEYNOTE-361 [24], pembrolizumab monotherapy did not improve OS compared with chemotherapy among patients aged ≥65 years (HR 1.15, 95% CI 0.84–1.59). Similarly, the reconstructed estimate from IMvigor130 [26] showed no OS advantage with atezolizumab monotherapy (HR 0.98, 95% CI 0.79–1.21). These findings do not support an OS benefit from single-agent ICI in the available ≥65-year subgroups. However, they do not establish whether chronological age modifies treatment efficacy, because formal treatment-by-age interaction analyses were not consistently available. More broadly, subgroup-specific HRs quantify treatment effects within the corresponding age-defined population, but do not determine whether those effects differ from, or are comparable to, those observed in younger patients. Exploratory data from EV-302 [29] further support the activity of enfortumab vedotin plus pembrolizumab in patients aged ≥75 years. In this subgroup, the objective response rate was 66.3%, closely resembling that observed in the overall trial population. Grade ≥3 adverse events occurred in 57.3% of patients, highlighting the need to balance efficacy against toxicity, functional status, and competing health risks in older patients. This individual age-specific safety estimate, however, does not allow comparison of tolerability across treatment classes or determination of whether toxicity differs according to age. Because age-specific safety, treatment discontinuation, dose modification, functional decline, and quality-of-life data were inconsistently reported across trials, the present analysis cannot define the overall benefit–risk profile of individual treatment strategies in patients aged ≥65 years. For patients without disease progression after platinum-based induction chemotherapy, avelumab maintenance remains clinically relevant. In the age-specific analysis of JAVELIN Bladder 100 [30], the OS benefit of avelumab was maintained across the age categories examined, and quality-adjusted time without symptoms or toxicity was prolonged by more than 4.5 months. However, maintenance data were summarized descriptively because the available evidence did not support a methodologically robust quantitative comparison across maintenance strategies in older patients.

Several limitations should be considered. All efficacy estimates were derived from trial-level age subgroup analyses that were generally exploratory and not powered to estimate treatment effects specifically in older patients. Age definitions were inconsistent across studies, and some estimates for patients aged ≥65 years were reconstructed from separate age strata. Moreover, the ≥65-year threshold was adopted as an operational category based on trial reporting and should not be interpreted as a geriatric definition. Chronological age alone does not capture frailty, comorbidity burden, renal function, performance status, physiological reserve, or competing health risks. Patients in their mid-to-late sixties may therefore differ substantially from frail patients or from those aged ≥75 years. Subgroup-specific baseline characteristics were largely unavailable, requiring use of overall intention-to-treat population characteristics for descriptive purposes. Consequently, the clinical and geriatric profile of the patients aged ≥65 years who contributed to the efficacy estimates could not be adequately characterized. The included trials also predominantly enrolled patients with preserved performance status and adequate organ function, limiting the generalizability of these findings to frail adults, patients with substantial comorbidity, and those with geriatric impairments. Consistent with these limitations, the GRADE certainty of evidence was rated as low for OS with first-line combination strategies and very low for PFS, ICI monotherapy, and maintenance strategies. Finally, formal treatment-by-age interaction estimates were not consistently reported across the included trials. Therefore, the present analysis cannot determine whether chronological age modifies the relative treatment effect. Comparisons of statistical significance between younger and older subgroups should not be interpreted as evidence of interaction. Future studies should report standardized age categories, include geriatric assessment measures, and provide age-specific data on efficacy, toxicity, treatment discontinuation, functional outcomes, and patient-reported outcomes.

## 5. Conclusions

First-line immune-based combinations were associated with improved OS in patients aged ≥65 years with advanced urothelial carcinoma. However, the magnitude of effect varied across treatment classes, and the substantial heterogeneity, prediction interval crossing the null value, and attenuation of the pooled estimate after exclusion of ADC plus ICI trials indicate that the overall pooled result should not be interpreted as evidence of a uniform benefit across therapeutic strategies. ADC plus ICI regimens produced the most favourable numerical estimates, but the available evidence does not support a formal efficacy ranking across treatment classes. The present findings characterize treatment effects within patients aged ≥65 years, but do not demonstrate that treatment efficacy is independent of chronological age or that age modifies treatment efficacy. These questions require direct comparison of treatment effects across age groups and formal treatment-by-age interaction analyses. The ≥65-year threshold should be interpreted as an operational trial-reporting category rather than as a geriatric definition. Therefore, the present results should not be extrapolated uncritically to frail patients, patients with substantial comorbidity or geriatric impairment, or those aged ≥75 years. Because age-specific toxicity and treatment-discontinuation data were insufficient for comparative analysis, these efficacy findings also do not establish the overall benefit–risk profile of individual treatment strategies. Treatment selection in older patients should integrate expected efficacy with toxicity, frailty, comorbidity burden, organ function, functional status, physiological reserve, and patient preferences. Future trials should adopt standardized age categories, prospectively incorporate geriatric assessment, and report age-specific efficacy, toxicity, treatment discontinuation, patient-reported outcomes, and treatment-by-age interaction analyses where appropriate.

## Figures and Tables

**Table 1:** Baseline characteristics of the included studies and patient populations.

Study Name	EXP ARM	Patients, *N*	≥65 Years Patients, *N* (%)	Median Age, Years (Range)	ECOG PS 0 (%)	ECOG PS 1 (%)	CIS Eligibility (%)	Visceral MTS (%)	Liver MTS (%)	Lymph-Node Only (%)	Best Response to First-Line (%)
Checkmate 901 [14,15]	Nivo + Ipi (arm A)	Exp: 221 Ctr: 224	Unk	Exp: 70 (48–87) Ctr: 71 (35–93)	Exp: 47.5% Ctr: 48.2%	Exp: 52.0% Ctr: 51.3%	NA **	Unk	Exp: 23% Ctr: 22%	Unk	NA
	Nivo + CT (arm C)	Exp: 304 Ctr 304	Exp: 154 (50.6%) Ctr: 156 (51.3%)	Exp: 65 (32–86) Ctr: 65 (35–85)	Exp: 53.3% Ctr: 53.3%	Exp: 46.0% Ctr: 56.7%	Exp: 100% Ctr: 100%	Unk	Exp: 21.1% Ctr: 21.1%	Unk	NA
KEYNOTE 361 [16]	Pem + CT	Exp: 351 Ctr: 301	Exp: 233 (66.4%) Ctr: 233 (77.4%)	Exp: 69 (62–75) Ctr: 69 (61–75)	Exp: 42.7% Ctr: 55.8%	Exp: 50.7% Ctr: 53.8%	Exp: 44% Ctr: 44%	Exp: 74% Ctr: 72%	Exp: 22% Ctr: 21%	Exp: 23% Ctr: 27%	NA
	Pem	Exp: 307 Ctr: 352	Exp: 198 (64.5%) Ctr: 233 (66.2%)	Exp: 68 (61–74) Ctr: 69 (61–75)	Exp: 43.6% Ctr: 47.7%	Exp: 48.2% Ctr: 46.0%	Exp: 45% Ctr: 44%	Exp: 78% Ctr: 72%	Exp: 21% Ctr: 21%	Exp: 21% Ctr: 27%	NA
EV-302 [19]	EV + Pem	Exp: 442 Ctr: 444	Exp *: 102 (23.1%) Ctr *: 108 (24.3%)	Exp: 69 (37–87) Ctr: 69 (22–91)	Exp: 52.7% Ctr: 48.4%	Exp: 46.1% Ctr: 48.6%	Exp: 54.3% Ctr: 54.5%	Exp: 71.9% Ctr: 71.6%	Exp: 22.6% Ctr: 22.3%	Exp: 23.3% Ctr: 23.4%	NA
DANUBE [17]	Durva	Exp: 346 Ctr: 344	Exp: 209 (60.4%) Ctr: 211 (61.3%)	Exp: 67 (60–73) Ctr: 68 (60–73)	Exp: 30.6% Ctr: 34.3%	Exp: 37.5% Ctr: 35.7%	Exp: 56% Ctr: 55%	Exp: 81% Ctr: 73%	Ext: 20% Ctr: 25%	Exp: 19% Ctr: 27%	NA
	Durva + Treme	Exp: 342 Ctr: 344	Exp: 205 (59.9%) Ctr: 211 (61.3%)	Exp: 68 (60–73) Ctr: 68 (60–73)	Exp: 33.9% Ctr: 34.3%	Exp: 35.6% Ctr: 35.7%	Exp: 56% Ctr: 55%	Exp: 76% Ctr: 73%	Exp: 22% Ctr: 25%	Exp: 23% Ctr: 27%	NA
IMvigor130 [18]	Atezo + CT	Exp: 451 Ctr: 400	Exp: 298 (66.0%) Ctr: 247 (61.7%)	Exp: 69 (62–75) Ctr: 67 (61–73)	Exp: 40.3% Ctr: 43.2%	Exp: 46.3% Ctr: 46.7%	Exp: 42% Ctr: 44%	Exp: 57% Ctr: 60%	Exp: 21% Ctr: 23%	Exp: 18% Ctr: 17%	NA
	Atezo	Exp: 362 Ctr: 400	Exp: 220 (60.7%) Ctr: 247 (61.7%)	Exp: 67 (62–74) Ctr: 67 (61–73)	Exp: 43.3% Ctr: 43.2%	Exp: 48.0% Ctr: 46.7%	Exp: 47% Ctr: 44%	Exp: 56% Ctr: 60%	Exp: 23% Ctr: 23%	Exp: 19% Ctr: 17%	NA
RC48-C016 [20]	DV + Tori	Exp: 243 Ctr: 241	Exp: 137 (56.4%) Ctr: 147 (61.0%)	Exp: 66 (39–84) Ctr: 67 (33–85)	Exp: 25.1% Ctr: 26.9%	Exp: 74.9% Ctr: 73.0%	Exp: 52.3% Ctr: 53.1%	Exp: 51% Ctr: 52.3%	Exp: 19.8% Ctr: 19.1%	Unk	NA
JAVELIN Bladder 100 [12]	Ave	Exp: 350 Ctr: 350	Exp: 221 (63.1%) Ctr: 243 (69.4%)	Exp: 73 (65–90) Ctr: 72 (65–89)	Exp: 60.9% Ctr: 60.3%	Exp: 38.9% Ctr: 38.9%	NA	Exp: 50% Ctr: 51.4%	Exp: 27% Ctr: 24.3%	NA	CR or PR Exp: 72.3% Ctr: 72% SD Exp: 27.7% Ctr: 28%
JAVELIN Bladder Medley [13]	Ave + SG	Exp: 74 Ctr: 37	NA	Exp: 70 (42–85) Ctr: 67 (53–89)	Exp: 68.9% Ctr: 45.9%	Exp: 31.1% Ctr: 54.1%	NA	Exp: 50% Ctr: 51.4%	Exp: 27% Ctr: 24.3%	NA	CR or PR Exp: 73% Ctr: 78.4% SD Exp: 25.7% Ctr: 21.6%

** Not applicable, as CheckMate 901 arm A enrolled cisplatin-ineligible patients only. * For EV-302, the reported baseline age category corresponds to patients aged ≥75 years, as baseline counts for patients aged ≥65 years were not reported.

**Table 2:** Overall and age-specific efficacy outcomes of the included studies.

Study Name (First Author, Year)	Treatment Arms	Median Follow-Up (mo)	Median OS Exp vs. Ctr (mo)	Median OS in ≥65 Years Patients Exp vs. Ctr (mo)	Median PFS (mo)	Median PFS in ≥65 Years Patients (mo)
Checkmate 901 (van der Heijden MS, 2023) [22,23]	Exp: Nivo + Ipi Ctr: Carboplatin + Gem	69.2	19.1 vs. 13.2 HR 0.79 (95% CI: 0.61–1.01)	Age 65–74 20.5 vs. 13.9 HR 0.80 (95% CI: 0.58–1.10) Age ≥ 75 12.3 vs. 14.5 HR 0.90 (95% CI: 0.63–1.30)	5.3 vs. 5.9 HR 0.90 (95% CI: 0.72–1.12)	Unk
	Exp: Nivo + Cisplatin + Gem Ctr: Cisplatin + Gem	33.6	21.7 vs. 18.9 HR 0.78 (95% CI: 0.63–0.96)	Age 65–74 HR 0.89 (95% CI: 0.63–1.26) Age ≥ 75 HR 0.86 (95% CI: 0.49–1.52)	7.9 vs. 7.6 HR 0.72 (95% CI: 0.59–0.88)	Age 65–74 HR 0.74 (95% CI: 0.54–1.02) Age ≥ 75 HR 0.60 (95% CI: 0.35–1.01)
KEYNOTE 361 (Powles T, 2021) [24]	Exp: Pem + Platinum + Gem Ctr: Platinum + Gem	31.7	17.0 vs. 14.3 HR 0.86 (95% CI: 0.72–1.02)	HR 0.88 (95% CI: 0.71–1.09)	8.3 vs. 7.1 HR 0.78 (95% CI: 0.65–0.93)	Unk
	Exp: Pem Ctr: Platinum + Gem	31.7	16.1 vs. 15.2 HR 0.92 (95% CI 0.77–1.11)	HR 1.15 (95% CI: 0.84–1.59)	Unk	Unk
EV-302 (Powles T, 2025) [27]	Exp: EV + Pem Ctr: Platinum + Gem	29.1	33.8 vs. 15.9 HR 0.51 (95% CI: 0.43–0.61)	27.1 vs. 14.6 HR 0.54 (0.44–0.67)	12.5 vs. 6.3 HR 0.48 (95% CI: 0.41–0.57)	12.3 vs. 6.2 HR 0.48 (95% CI: 0.39–0.59)
DANUBE (Powles T, 2020) [25]	Exp: Durva Ctr: Platinum + Gem	41.2	13.2 vs. 12.1 HR 0.99 (95% CI. 0.83–1.17)	Unk	Unk	Unk
	Exp: Durva + Treme Ctr: Platinum + Gem	41.2	15.1 vs. 12.1 HR 0.85 (95% CI: 0.72–1.02)	13.5 vs. 11.8 HR 0.98 (95% CI: 0.79–1.23)	Unk	Unk
IMvigor130 (Bamias A, 2024) [26]	Exp: Atezo + Platinum + Gem Ctr: Platinum + Gem	13.4	16.1 vs. 13.4 HR 0.85 (95% CI: 0.73–1.00)	Age 65–80 17.2 vs. 14.6 HR 0.81 (95% CI: 0.66–1.00) Age ≥ 80 12.2 vs. 11.3 mo HR 0.94 (95% CI: 0.53–1.66)	8.2 vs. 6.3 HR 0.82 (95% CI: 0.70–0.96)	7.5 vs. 6.3 HR 0.80 (95% CI: 0.66–0.97)
	Exp: Atezo Ctr: Platinum + Gem	13.4	15.2 vs. 13.3 HR 0.98 (95% CI: 0.82–1.16)	Age 65–80 14.7 vs. 14.6 HR 1.00 (95% CI: 0.80–1.26) Age ≥ 80 14.9 vs. 11.3 HR 0.82 (95% CI: 0.4–1.57)	Unk	Unk
RC48-C016 (Sheng X, 2025) [28]	Exp: DV + Tori Ctr: Platinum + Gem	18.2	31.5 vs. 16.9 HR 0.54 (95% CI: 0.41–0.73)	23.2 vs. 16.5 HR 0.62 (95% CI: 0.43–0.89)	13.1 vs. 6.5 HR 0.36 (95% CI: 0.28–0.46)	14.4 vs. 5.9 HR 0.30 (95% CI: 0.21–0.43)
JAVELIN Bladder 100 (Powles T, 2020) [20]	Exp: Ave + BSC Ctr: BSC	38.0	21.4 vs. 14.3 HR 0.69 (95% CI: 0.56–0.86)	26.1 vs. 15.5 HR 0.70 (95% CI: 0.56–0.89)	3.7 vs. 2.0 HR 0.62 (95% CI: 0.52–0.75)	7.4 vs. 2.5 HR 0.46 (95% CI: 0.37–0.57)
Javelin Bl Medley (Hoffman-Censits J, 2025) [21]	Exp: Ave + SG Ctr: Ave	10.9	NR vs. 23.75 HR Unk	Unk	11.2 vs. 3.7 HR 0.49 (95% CI: 0.31–0.76)	9.2 vs. 5.7 HR 0.68 (95% CI: 0.41–1.14)

## Data Availability

No new data were created or analyzed in this study. Data sharing is not applicable to this article.

## Supplementary Materials

The following supporting information can be downloaded at https://www.mdpi.com/article/10.3390/cancers18182924/s1, Section S1: 27 item PRISMA Checklist 2020. Section S2: PICO (Population, Intervention, Comparison, and Outcome). Section S3: Deviations from Initial Protocol. Figure S1: Risk-of-bias assessment of the included studies. Traffic-light plot showing domain-level and overall risk-of-bias judgements according to the Cochrane Risk of Bias 2 tool. Most randomized comparisons were judged to be at low overall risk of bias. Green circles indicate low risk of bias, yellow circles indicate some concerns, and red circles indicate high risk of bias. Abbreviations: D1, bias arising from the randomization process; D2, bias due to deviations from intended interventions; D3, bias due to missing outcome data; D4, bias in measurement of the outcome; D5, bias in selection of the reported result; RoB 2, Risk of Bias 2. Figure S2: Overall survival according to trial design in patients aged ≥65 years with advanced urothelial carcinoma. Forest plot of hazard ratios for overall survival among patients aged ≥65 years, stratified by multi-arm versus standard two-arm trial design. CheckMate 901 was not classified as a multi-arm trial for this analysis because arms A and C included separate control cohorts drawn from clinically distinct populations with different cisplatin-eligibility criteria. Study-specific estimates were pooled using random-effects models with restricted maximum likelihood estimation and Hartung-Knapp adjustment. Squares represent study-specific estimates, with their size proportional to study weight; horizontal lines indicate 95% confidence intervals, and diamonds represent pooled estimates. Abbreviations: Atezo, atezolizumab; CI, confidence interval; CT, chemotherapy; df, degrees of freedom; Durva, durvalumab; DV, disitamab vedotin; EV, enfortumab vedotin; HK, Hartung-Knapp; HR, hazard ratio; Ipi, ipilimumab; Nivo, nivolumab; OS, overall survival; Pembro, pembrolizumab; Tori, toripalimab; Treme, tremelimumab; χ^2^, chi-square statistic; τ^2^, between-study variance. Figure S3: Sensitivity analysis of overall survival after exclusion of antibody-drug conjugate plus immune checkpoint inhibitor combinations. Forest plot of hazard ratios for overall survival among patients aged ≥65 years treated with dual immune checkpoint inhibitor combinations or immune checkpoint inhibitors plus chemotherapy versus platinum-based chemotherapy. Study-specific estimates were pooled using random-effects models with restricted maximum likelihood estimation and Hartung-Knapp adjustment. Squares represent study-specific estimates, with their size proportional to study weight; horizontal lines indicate 95% confidence intervals, and diamonds represent pooled estimates. Abbreviations: ADC, antibody-drug conjugate; Atezo, atezolizumab; CI, confidence interval; CT, chemotherapy; df, degrees of freedom; Durva, durvalumab; HK, Hartung-Knapp; HR, hazard ratio; ICI, immune checkpoint inhibitor; Ipi, ipilimumab; Nivo, nivolumab; OS, overall survival; Pembro, pembrolizumab; Treme, tremelimumab; χ^2^, chi-square statistic; τ^2^, between-study variance. Table S1: Leave-one-out sensitivity analysis for overall survival in patients aged ≥65 years included in the primary first-line combination-versus-platinum-based chemotherapy meta-analysis. Each row reports the pooled hazard ratio after sequential exclusion of the indicated study or comparison; the row “None” corresponds to the primary analysis. Estimates were obtained using a random-effects model with restricted maximum likelihood estimation and Hartung–Knapp confidence intervals. CI, confidence interval; HR, hazard ratio. Table S2: GRADE summary of certainty of evidence for age-specific efficacy outcomes in patients aged ≥65 years. Certainty of evidence was assessed using the GRADE approach. Ratings refer to treatment-effect estimates within the ≥65-year subgroup and should not be interpreted as evidence of treatment-effect modification by age. CI, confidence interval; GRADE, Grading of Recommendations Assessment, Development and Evaluation; HR, hazard ratio; ICI, immune checkpoint inhibitor; OS, overall survival; PFS, progression-free survival.

## Abbreviations

The following abbreviations are used in this manuscript:

ADC	Antibody–drug conjugate
Atezo	Atezolizumab
Ave	Avelumab
BSC	Best supportive care
CI	Confidence interval
CIS	Cisplatin
CR	Complete response
CT	Chemotherapy
Ctr	Control
Durva	Durvalumab
DV	Disitamab vedotin
ECOG PS	Eastern Cooperative Oncology Group performance status
EV	Enfortumab vedotin
Exp	Experimental
Gem	Gemcitabine
ICI	Immune checkpoint inhibitor
Ipi	Ipilimumab
MTS	Metastases
Nivo	Nivolumab
PD-1	Programmed cell death protein 1
PD-L1	Programmed death-ligand 1
Pem	Pembrolizumab
PFS	Progression-free survival
SG	Sacituzumab govitecan
Tori	Toripalimab
Treme	Tremelimumab
UC	Urothelial carcinoma
Unk	Unknown
