# Supplementary material for: First-Line Immune Checkpoint Inhibitors in Older Patients with Advanced Urothelial Carcinoma: A Systematic Review and Meta-Analysis
Source: Cancers (Basel). 2026 Sep 9;18(18):2924. doi: 10.3390/cancers18182924 (PMC13604697; doi:10.3390/cancers18182924)
Supplement: Supplementary file 1 [file cancers-18-02924-s001.zip › cancers-4500169-supplementary.pdf]

Supplementary Materials

Supplementary Section S1. 27 item PRISMA Checklist 2020

Section and Topic	Item #	Checklist item	Location where item is reported
**TITLE**			
Title	1	Identify the report as a systematic review.	Title
**ABSTRACT**			
Abstract	2	See the PRISMA 2020 for Abstracts checklist.	Abstract
**INTRODUCTION**			
Rationale	3	Describe the rationale for the review in the context of existing knowledge.	1.Introduction
Objectives	4	Provide an explicit statement of the objective(s) or question(s) the review addresses.	1.Introduction<br>2.1 Protocol registration and endpoints
**METHODS**			
Eligibility criteria	5	Specify the inclusion and exclusion criteria for the review and how studies were grouped for the syntheses.	2.2 Eligibility criteria
Information sources	6	Specify all databases, registers, websites, organisations, reference lists and other sources searched or consulted to identify studies. Specify the date when each source was last searched or consulted.	2.3 Search strategy and study selection<br>Supplementary Section S3
Search strategy	7	Present the full search strategies for all databases, registers and websites, including any filters and limits used.	2.3 Search strategy and study selection
Selection process	8	Specify the methods used to decide whether a study met the inclusion criteria of the review, including how many reviewers screened each record and each report retrieved, whether they worked independently, and if applicable, details of automation tools used in the process.	2.2 Eligibility criteria<br>2.4 Data Extraction and Risl o Bias
Data collection process	9	Specify the methods used to collect data from reports, including how many reviewers collected data from each report, whether they worked independently, any processes for obtaining or confirming data from study investigators, and if applicable, details of automation tools used in the process.	2.4 Data Extraction and Risl o Bias
Data items	10a	List and define all outcomes for which data were sought. Specify whether all results that were compatible with each outcome domain in each study were sought (e.g. for all measures, time points, analyses), and if not, the methods used to decide which results to collect.	2.1 Protocol registration and endpoints<br>2.2 Eligibility criteria
	10b	List and define all other variables for which data were sought (e.g. participant and intervention characteristics, funding sources). Describe any assumptions made about any missing or unclear information.	2.1 Protocol registration and endpoints<br>2.2 Eligibility criteria
Study risk of bias assessment	11	Specify the methods used to assess risk of bias in the included studies, including details of the tool(s) used, how many reviewers assessed each study and whether they worked independently, and if applicable, details of automation tools used in the process.	2.4 Data Extraction and Risk of Bias

Effect measures	12	Specify for each outcome the effect measure(s) (e.g. risk ratio, mean difference) used in the synthesis or presentation of results.	2.5 Statistical data analysis
Synthesis methods	13a	Describe the processes used to decide which studies were eligible for each synthesis (e.g. tabulating the study intervention characteristics and comparing against the planned groups for each synthesis (item #5)).	2.2 Eligibility criteria<br>2.3 Search strategy and study selection
	13b	Describe any methods required to prepare the data for presentation or synthesis, such as handling of missing summary statistics, or data conversions.	2.5 Statistical data analysis
	13c	Describe any methods used to tabulate or visually display results of individual studies and syntheses.	2.4 Data Extraction and Risk of Bias<br>2.5 Statistical data analysis
	13d	Describe any methods used to synthesize results and provide a rationale for the choice(s). If meta-analysis was performed, describe the model(s), method(s) to identify the presence and extent of statistical heterogeneity, and software package(s) used.	2.5 Statistical data analysis<br>2.6 Sensitivity Analyses
	13e	Describe any methods used to explore possible causes of heterogeneity among study results (e.g. subgroup analysis, meta-regression).	2.5 Statistical data analysis<br>2.6 Sensitivity Analyses
	13f	Describe any sensitivity analyses conducted to assess robustness of the synthesized results.	2.5 Statistical data analysis
Reporting bias assessment	14	Describe any methods used to assess risk of bias due to missing results in a synthesis (arising from reporting biases).	2.5 Statistical data analysis
Certainty assessment	15	Describe any methods used to assess certainty (or confidence) in the body of evidence for an outcome.	2.5 Statistical data analysis
**RESULTS**			
Study selection	16a	Describe the results of the search and selection process, from the number of records identified in the search to the number of studies included in the review, ideally using a flow diagram.	3.1 Study selection
	16b	Cite studies that might appear to meet the inclusion criteria, but which were excluded, and explain why they were excluded.	3.2 Study and patient characteristics
Study characteristics	17	Cite each included study and present its characteristics.	3.2 Study and patient characteristics<br>Table 2
Risk of bias in studies	18	Present assessments of risk of bias for each included study.	3.3 RoB<br>Supplementary Figure S1
Results of individual studies	19	For all outcomes, present, for each study: (a) summary statistics for each group (where appropriate) and (b) an effect estimate and its precision (e.g. confidence/credible interval), ideally using structured tables or plots.	Table 1<br>Table 2
Results of syntheses	20a	For each synthesis, briefly summarise the characteristics and risk of bias among contributing studies.	Supplementary Figure S1
	20b	Present results of all statistical syntheses conducted. If meta-analysis was done, present for each the summary estimate and its precision (e.g. confidence/credible interval) and measures of statistical heterogeneity. If comparing groups, describe the direction of the effect.	3.4.1 First-line treatment and OS<br>3.4.2 First-line treatment and PFS<br>3.4.3 Maintenance treatment: descriptive synthesis

	20c	Present results of all investigations of possible causes of heterogeneity among study results.	3.4.4 Exploratory and Sensitivity analyses<br>Supplementary Figure S2<br>Supplementary Figure S3
	20d	Present results of all sensitivity analyses conducted to assess the robustness of the synthesized results.	3.4.4 Exploratory and Sensitivity analyses<br>Supplementary Figure S2<br>Supplementary Figure S3<br>Supplementary Table S1
Reporting biases	21	Present assessments of risk of bias due to missing results (arising from reporting biases) for each synthesis assessed.	3.3 RoB
Certainty of evidence	22	Present assessments of certainty (or confidence) in the body of evidence for each outcome assessed.	3.4.1 First-line treatment and OS<br>3.4.2 First-line treatment and PFS<br>3.4.3 Maintenance treatment: descriptive synthesis
**DISCUSSION**			
Discussion	23a	Provide a general interpretation of the results in the context of other evidence.	4.Discussion
	23b	Discuss any limitations of the evidence included in the review.	4.Discussion
	23c	Discuss any limitations of the review processes used.	4.Discussion
	23d	Discuss implications of the results for practice, policy, and future research.	4.Discussion
**OTHER INFORMATION**			
Registration and protocol	24a	Provide registration information for the review, including register name and registration number, or state that the review was not registered.	2.1 Protocol registration and endpoints<br>PROSPERO Protocol
	24b	Indicate where the review protocol can be accessed, or state that a protocol was not prepared.	2.1 Protocol registration and endpoints<br>PROSPERO Protocol
	24c	Describe and explain any amendments to information provided at registration or in the protocol.	2.1 Protocol registration and endpoints<br>PROSPERO Protocol<br>Supplementary Section S2.
Support	25	Describe sources of financial or non-financial support for the review, and the role of the funders or sponsors in the review.	Funding
Competing interests	26	Declare any competing interests of review authors.	Conflict of interest
Availability of data, code and other materials	27	Report which of the following are publicly available and where they can be found: template data collection forms; data extracted from included studies; data used for all analyses; analytic code; any other materials used in the review.	The following materials are publicly available as Supplementary Files:<br>- Data extraction forms used during the review process (Table 1 and 2);<br>- Extracted data from included studies, including hazard ratios and confidence intervals for visceral disease subgroups (Table 2);

			- Analytic code used for network meta-analysis and graphical outputs (available upon request");<br>- Network structure and PRISMA flowchart (Figure 1).<br>All materials used in the review are available upon request from the corresponding author.

Supplementary Section S2. PICO (Population, Intervention, Comparison, and Outcome)

- Population (P): Older adult patients, defined by trial-specific subgroups of ≥ 65 years of age, diagnosed with locally advanced or metastatic UC that was previously untreated.
- Intervention (I): Systemic immune-based therapies; this comprised monotherapy or combination strategies with standard CT, antibody-drug conjugates (ADC) or other ICI).
- Comparison (C): Protocol-defined standard of care regimens, specifically platinum-based CT (i.e. Cisplatin or Carboplatin + Gemcitabine).
- Outcome (O): OS and PFS, provided that HRs and 95% CIs were explicated or calculable for the older age subpopulation.

Supplementary Section S3. Deviations from Initial Protocol

After protocol registration, selected methodological amendments were made to ensure that the final analyses remained clinically coherent and methodologically appropriate for the available evidence:

- The original protocol included the term "maintenance" in the title and scope. This word was subsequently removed to better reflect the final focus of the analysis on first-line systemic treatment strategies.
- The planned network meta-analysis was replaced by a pairwise meta-analysis. This modification was made because the available subgroup-level data did not support a reliable connected network of clinically comparable treatment nodes. A pairwise framework was therefore considered more appropriate and statistically transparent for the available evidence.
- Maintenance trials were excluded from the quantitative synthesis. JAVELIN Bladder Medley evaluated avelumab-based combinations versus avelumab plus best supportive care; however, the control arm was extended per protocol using propensity score-weighted data derived from the avelumab arm of JAVELIN Bladder 100. This design feature violated the assumption of inter-trial independence and precluded a valid formal indirect comparison. Accordingly, no quantitative indirect comparison involving maintenance strategies was performed.

SUPPLEMENTARY FIGURES

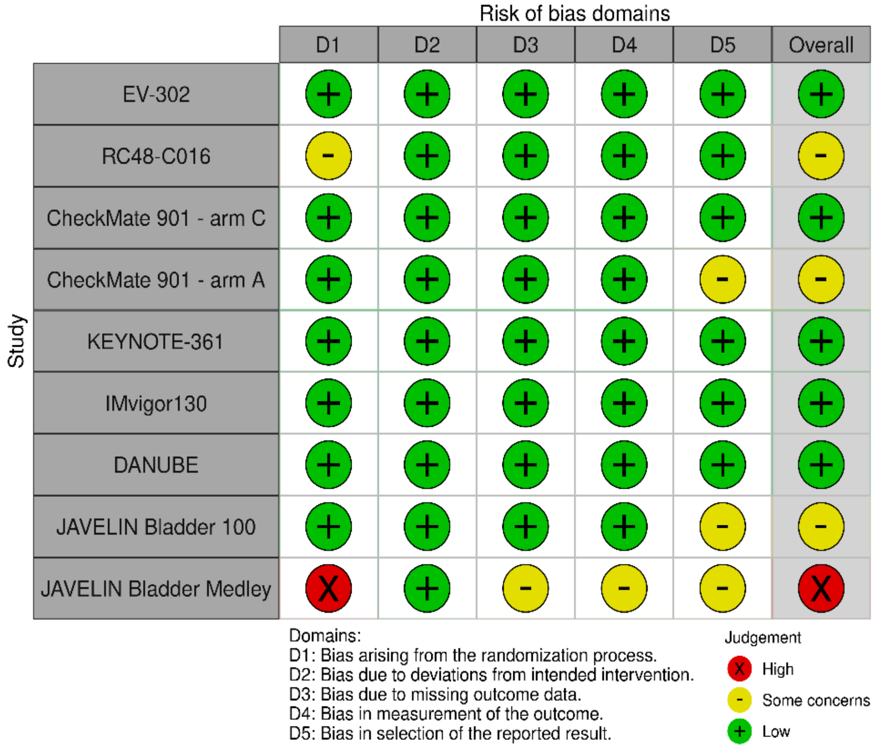

Supplementary Figure S1. Risk-of-bias assessment of the included studies. Traffic-light plot showing domain-level and overall risk-of-bias judgements according to the Cochrane Risk of Bias 2 tool. Most randomized comparisons were judged to be at low overall risk of bias. Green circles indicate low risk of bias, yellow circles indicate some concerns, and red circles indicate high risk of bias. Abbreviations: D1, bias arising from the randomization process; D2, bias due to deviations from intended interventions; D3, bias due to missing outcome data; D4, bias in measurement of the outcome; D5, bias in selection of the reported result; RoB 2, Risk of Bias 2.

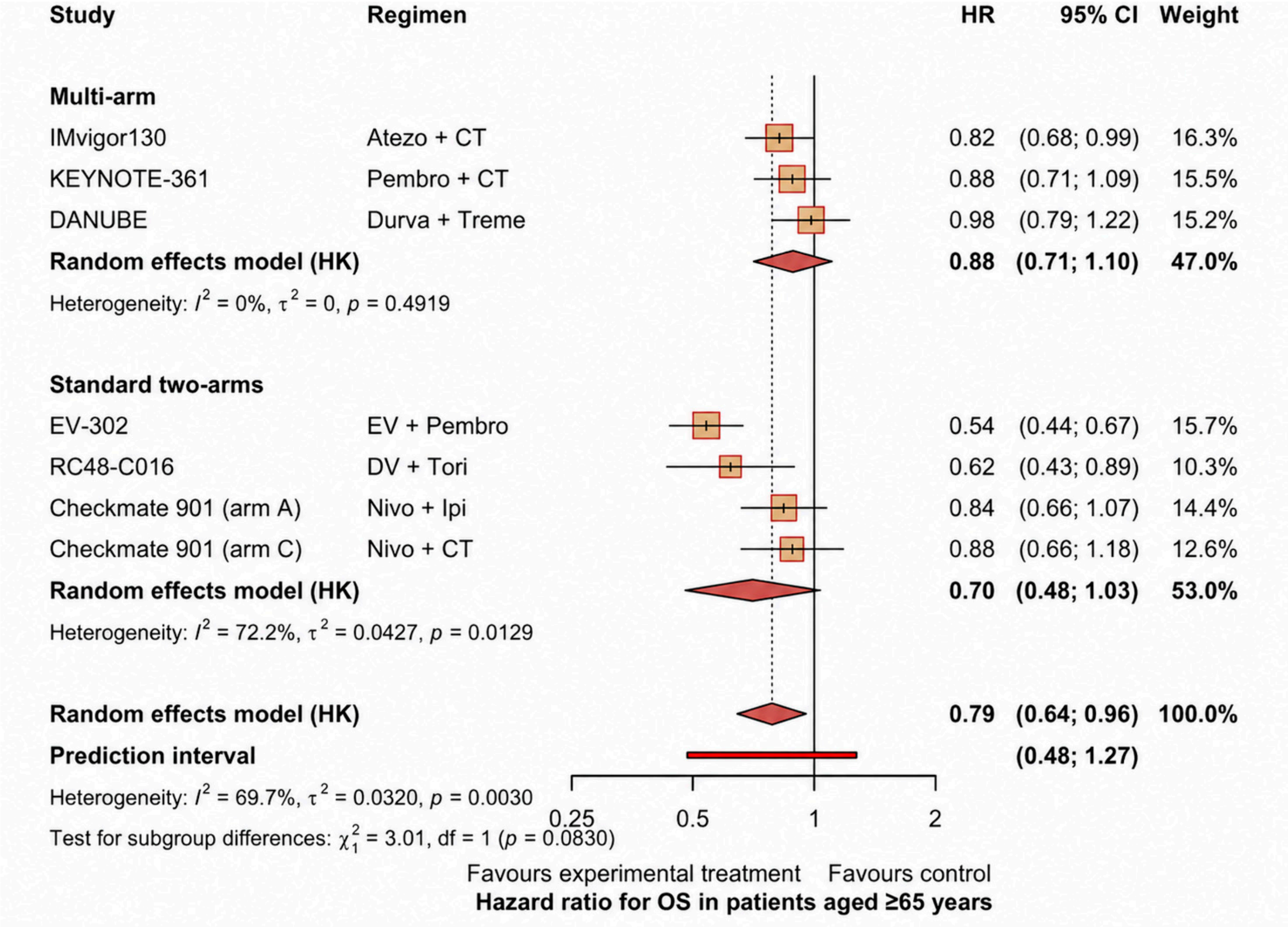

Supplementary Figure S2. Overall survival according to trial design in patients aged ≥65 years with advanced urothelial carcinoma. Forest plot of hazard ratios for overall survival among patients aged ≥65 years, stratified by multi-arm versus standard two-arm trial design. CheckMate 901 was not classified as a multi-arm trial for this analysis because arms A and C included separate control cohorts drawn from clinically distinct populations with different cisplatin-eligibility criteria. Study-specific estimates were pooled using random-effects models with restricted maximum likelihood estimation and Hartung-Knapp adjustment. Squares represent study-specific estimates, with their size proportional to study weight; horizontal lines indicate 95% confidence intervals, and diamonds represent pooled estimates. Abbreviations: Atezo, atezolizumab; CI, confidence interval; CT, chemotherapy; df, degrees of freedom; Durva, durvalumab; DV, disitamab vedotin; EV, enfortumab vedotin; HK, Hartung-Knapp; HR, hazard ratio; Ipi, ipilimumab; Nivo, nivolumab; OS, overall survival; Pembro, pembrolizumab; Tori, toripalimab; Treme, tremelimumab; χ^2, chi-square statistic; τ^2, between-study variance.

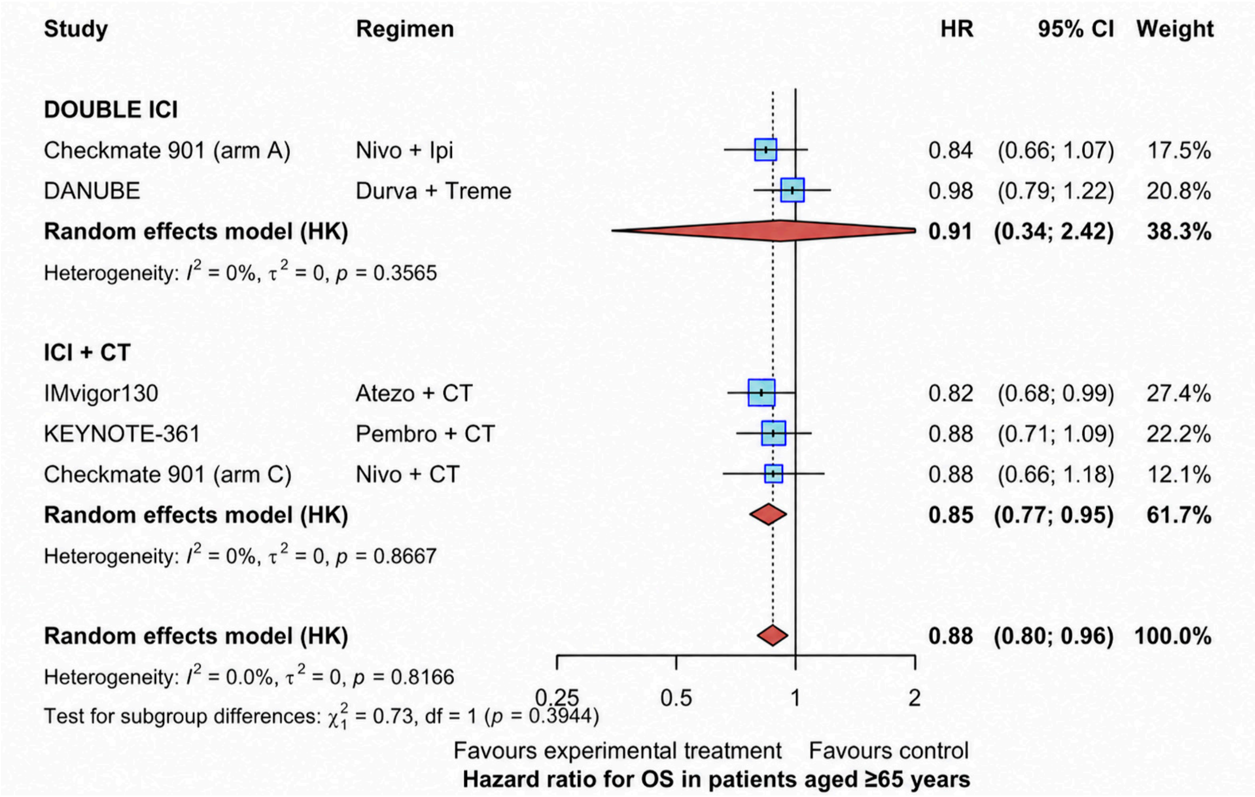

Supplementary Figure S3. Sensitivity analysis of overall survival after exclusion of antibody-drug conjugate plus immune checkpoint inhibitor combinations. Forest plot of hazard ratios for overall survival among patients aged ≥65 years treated with dual immune checkpoint inhibitor combinations or immune checkpoint inhibitors plus chemotherapy versus platinum-based chemotherapy. Study-specific estimates were pooled using random-effects models with restricted maximum likelihood estimation and Hartung-Knapp adjustment. Squares represent study-specific estimates, with their size proportional to study weight; horizontal lines indicate 95% confidence intervals, and diamonds represent pooled estimates. Abbreviations: ADC, antibody-drug conjugate; Atezo, atezolizumab; CI, confidence interval; CT, chemotherapy; df, degrees of freedom; Durva, durvalumab; HK, Hartung-Knapp; HR, hazard ratio; ICI, immune checkpoint inhibitor; Ipi, ipilimumab; Nivo, nivolumab; OS, overall survival; Pembro, pembrolizumab; Treme, tremelimumab; χ^2, chi-square statistic; τ^2, between-study variance.

SUPPLEMENTARY TABLES

Supplementary Table S1. Leave-one-out sensitivity analysis for overall survival in patients aged ≥65 years included in the primary first-line combination-versus-platinum-based chemotherapy meta-analysis. Each row reports the pooled hazard ratio after sequential exclusion of the indicated study or comparison; the row "None" corresponds to the primary analysis. Estimates were obtained using a random-effects model with restricted maximum likelihood estimation and Hartung–Knapp confidence intervals. CI, confidence interval; HR, hazard ratio.

**Study excluded**	**HR (95% CI)**	**p-value**	**I^2 (%)**	**τ^2**
None	0.79 (0.64–0.96)	0.025	69.7	0.032
KEYNOTE-361	0.77 (0.60–0.98)	0.038	73.1	0.038
EV-302	0.85 (0.75–0.97)	0.023	0.0	0.000
DANUBE	0.75 (0.61–0.94)	0.022	67.5	0.029
RC48-C016	0.81 (0.65–1.01)	0.055	72.3	0.033
Checkmate 901 (arm A)	0.78 (0.61–0.99)	0.045	74.4	0.040
Checkmate 901 (arm C)	0.77 (0.61–0.98)	0.039	74.0	0.038
IMvigor130	0.78 (0.61–1.00)	0.048	74.5	0.042

Supplementary Table S2. GRADE summary of certainty of evidence for age-specific efficacy outcomes in patients aged ≥65 years. Certainty of evidence was assessed using the GRADE approach. Ratings refer to treatment-effect estimates within the ≥65-year subgroup and should not be interpreted as evidence of treatment-effect modification by age. CI, confidence interval; GRADE, Grading of Recommendations Assessment, Development and Evaluation; HR, hazard ratio; ICI, immune checkpoint inhibitor; OS, overall survival; PFS, progression-free survival.

Outcome	No. of trials/comparisons	Relative Effect (95% CI)	Certainty of Evidence (GRADE)	Reasons for Downgrading
**Overall Survival (OS)**<br>*First-line combinations vs Chemotherapy*	7 trials	HR 0.79<br>(0.64 – 0.96)	⊕⊕○○<br>Low	**Inconsistency** (Serious): high heterogeneity, wide prediction interval crossing the null value;<br>**Indirectness** (Serious): exploratory subgroup estimates.
**Overall Survival (OS)**<br>*ICI monotherapy vs Chemotherapy*	2 trials	Descriptive, no significant OS benefit	⊕○○○<br>Very low	**Indirectness** (Serious): exploratory subgroup reporting;<br>**Imprecision** (Very Serious): limited number of estimates
**Progression-Free Survival (PFS)**<br>*First-line combinations vs Chemotherapy*	4 trials	HR 0.54<br>(0.27 – 1.08)	⊕○○○<br>Very low	**Inconsistency** (Serious): high heterogeneity;<br>**Indirectness** (Serious): exploratory subgroup estimates;<br>**Imprecision** (Serious): wide confidence interval crossing the null value.
**Maintenance treatment**	2 trials	Descriptive synthesis	⊕○○○<br>Very low	**Risk of Bias/Indirectness** (Very Serious): methodological limitations and non-independent external control data precluded indirect comparison;<br>**Imprecision** (Serious): no consistent age-specific OS data.
